# Analysis Tools for Large Connectomes

**DOI:** 10.3389/fncir.2018.00085

**Published:** 2018-10-15

**Authors:** Louis K. Scheffer

**Affiliations:** HHMI, Janelia Campus, Ashburn, VA, United States

**Keywords:** analysis of connectomes, EM reconstruction, neural circuits, neural simulation, reproducibility

## Abstract

New reconstruction techniques are generating connectomes of unprecedented size. These must be analyzed to generate human comprehensible results. The analyses being used fall into three general categories. The first is interactive tools used during reconstruction, to help guide the effort, look for possible errors, identify potential cell classes, and answer other preliminary questions. The second type of analysis is support for formal documents such as papers and theses. Scientific norms here require that the data be archived and accessible, and the analysis reproducible. In contrast to some other “omic” fields such as genomics, where a few specific analyses dominate usage, connectomics is rapidly evolving and the analyses used are often specific to the connectome being analyzed. These analyses are typically performed in a variety of conventional programming language, such as Matlab, R, Python, or C++, and read the connectomic data either from a file or through database queries, neither of which are standardized. In the short term we see no alternative to the use of specific analyses, so the best that can be done is to publish the analysis code, and the interface by which it reads connectomic data. A similar situation exists for archiving connectome data. Each group independently makes their data available, but there is no standardized format and long-term accessibility is neither enforced nor funded. In the long term, as connectomics becomes more common, a natural evolution would be a central facility for storing and querying connectomic data, playing a role similar to the National Center for Biotechnology Information for genomes. The final form of analysis is the import of connectome data into downstream tools such as neural simulation or machine learning. In this process, there are two main problems that need to be addressed. First, the reconstructed circuits contain huge amounts of detail, which must be intelligently reduced to a form the downstream tools can use. Second, much of the data needed for these downstream operations must be obtained by other methods (such as genetic or optical) and must be merged with the extracted connectome.

## 1. Introduction

A connectome is a detailed description of a neural circuit, including the neurons and the synaptic connections between them. New and improved reconstruction techniques, using electron microscopy(EM) (Chklovskii et al., 2010), optical labeling (Lichtman et al., 2008), or sequencing (Zador et al., 2012), are generating connectomes of unprecedented size. These must be analyzed to generate human comprehensible results and provide input to downstream tools. There are at least three very different use cases. The first is interactive analysis, used during the reconstruction itself. Next, there is formal analysis, for reports, papers, and proceedings. Finally, connectomes are used as input to further stages of analysis, such as simulation and machine learning algorithms.

Each of these use cases is rapidly evolving. The increased scale of reconstructions requires new interactive analysis methods for efficiency and quality control. The more formal analyses used so far are often specific to the connectome being analyzed. For example, the analyses used for extremely stereotyped circuits, such as the fly's optic lobe, are very different than the analyses used for the apparently random wiring of portions of olfactory systems. Finally, the usage of connectomes as input to further tools, such as simulation, is just beginning. It is not yet clear what the requirements are.

Analysis of connectomes is likely to follow the path of analysis of genomes. Initially, genomes were difficult to acquire, and the same group that did the acquisition did the analysis. But as the technology for sequencing improved, analysis became the limiting step. Groups that acquired genomes could no longer analyze all the data they collected, and conversely, many of the scientists who analyze genomes had no hand in the data collection. This same transition will likely happen in connectomics. One difference, however, is that connectomics has a much larger variety in the form, function, and usage of its analyses. This differs from genomics, where a few specific forms of analysis dominate the usage, as exemplified by the Basic Local Sequence Alignment Tool, or BLAST (Altschul et al., 1990).

## 2. Previous work

There is another usage of “Connectome,” that refers to the connections between regions of the brain, and not detailed connections between neurons. These apply to much larger animals where detailed neural reconstruction is not yet possible. This paper does not cover analysis of such connectomes, which has its own literature (Sporns, 2003; He et al., 2011; Kaiser, 2011; Leergaard et al., 2012; Xia et al., 2013).

At its heart, a connectome is a directed graph. Since graphs are useful representations in many science and engineering tasks, there has been considerable research into specific tasks on graphs, such as partitioning (Kernighan and Lin, 1970; Pothen et al., 1990; Karypis and Kumar, 1998), clustering (Hartuv and Shamir, 2000; Brandes et al., 2003; White and Smyth, 2005), finding cliques (Everett and Borgatti, 1998), finding patterns (Kuramochi and Karypis, 2005), finding small motifs (Itzkovitz and Alon, 2005) and so on. Only some of these techniques have been applied to connectomes, and it is not clear which, if any, can provide useful answers to practical biology problems.

One challenge with connectomes is that the connectomes are “fuzzy,” meaning every instance of a common sub-graph is slightly different. This means that some well-known graph and subgraph matching algorithms (such as Ullmann, 1976), particularly those based on graph invariants (Corneil and Kirkpatrick, 1980), may not work well when applied to connectomes. Conversely, algorithms designed to cope with errors, such as (Messmer and Bunke, 1998), are more likely to be applicable.

“ConnectomeExplorer” (Beyer et al., 2013) is an integrated tool, intended to solve many of the problems indicated in this article. It includes its own visualization tools and analysis language. However, it does not appear to have been used in any of the major connectome analysis efforts, likely because familiarity with conventional tools such as Matlab has outweighed the advantages of a new tool with its corresponding learning curve.

## 3. Discovery

Currently, there are three main use cases for connectomes, here called “discovery,” “formal,” and “input.”

“Discovery” involves inspecting the connectome for interesting features. These tools are typically fast and graphical in nature, and must work with the approximate connectomes that exist as reconstruction progresses. They are often built into the reconstruction tools, and are used to look at reconstruction concerns and ordering, as well as generate science results as early in the reconstruction process as practical. Examples include connectivity tables of various kinds, plot of connections as a function of graph connectivity or distance from the root of the neuron, and comparisons of seemingly similar neurons. In this paper, we look at tools used during past reconstructions, those being used currently in the still larger reconstructions in process, and those we think will be needed in the future.

**Figure 1:**
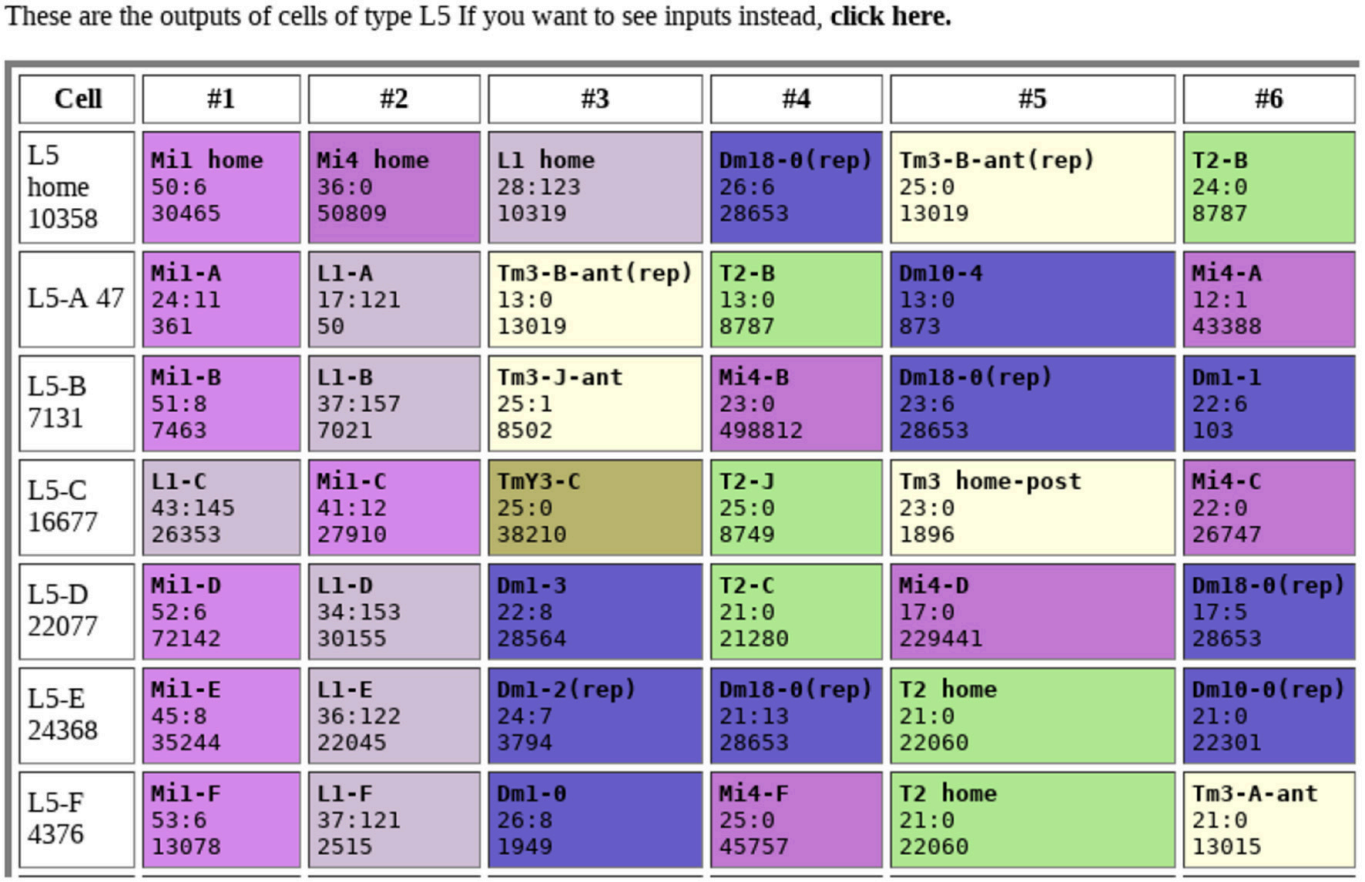
Example of connection table. Each row shows the connections to a single neuron, sorted by synapse count. Each box shows the identity of the connected neuron, the synapse count in both directions (separated by a “:”), and the internal identifier of the connected neuron. Colors are arbitrary, but all cells of the same type share the same color. Data from Takemura et al. (2015).

Tables of connections are one of the most obvious outputs. Typically, these show the upstream and downstream neurons, sorted by strength, as shown in Figure 1. Color coding makes connection patterns more obvious. Comparing rows shows the differences between neurons with similar names or types.

**Figure 2:**
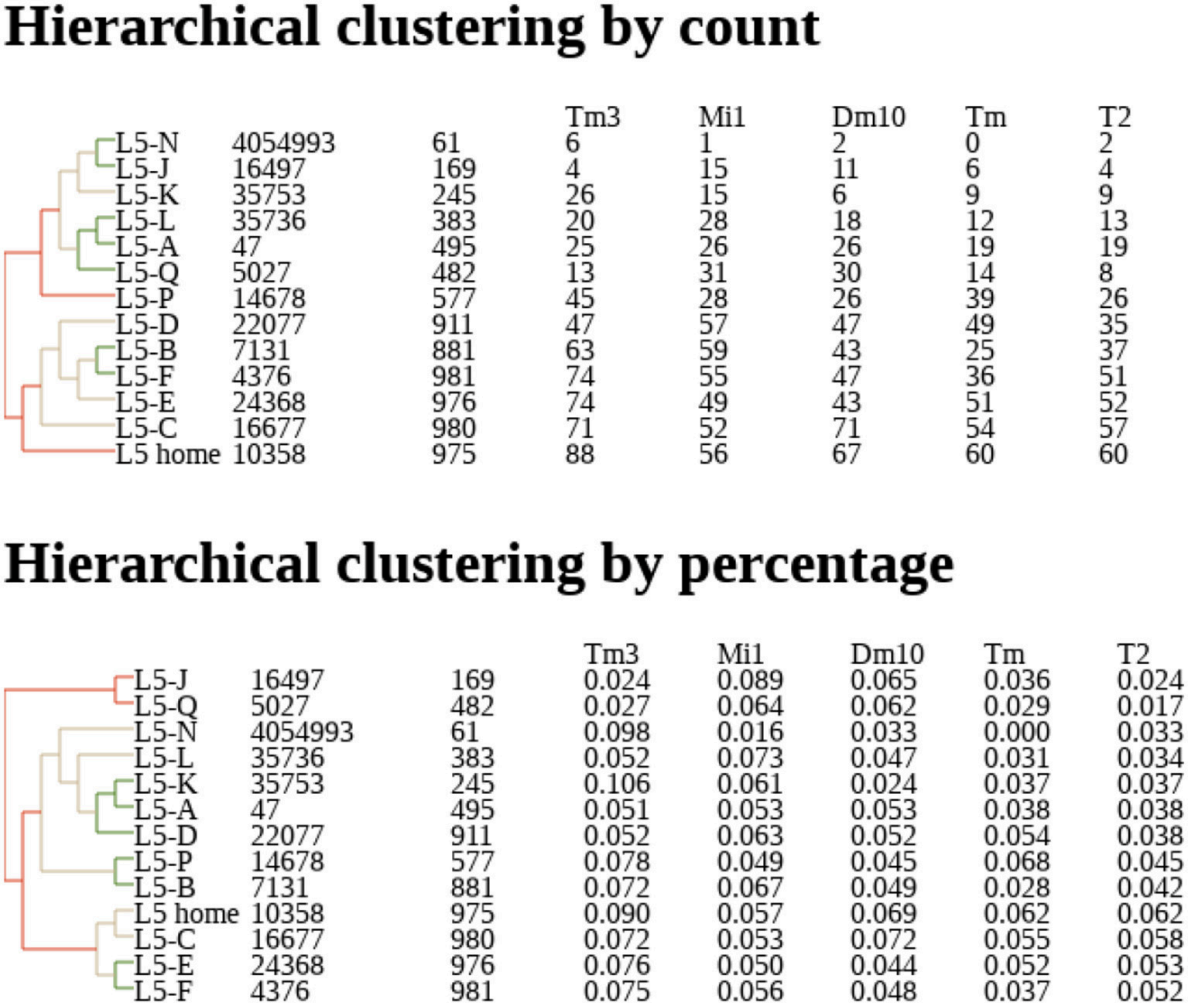
Example of dendrogram describing clustering of neurons by their connectivity, based on their proximity in N dimensional connectivity space, where N is the number of cell types to which this neuron is connected. Coordinates in this space are determined by synapse counts **(Top)** or percentage of input **(Bottom)**. Counts and percentages shown for the five most strongly connected types. Data from Takemura et al. (2015).

Dendrograms are another natural representation. Nervous systems often contain many similar cells, often referred to a “cell type.” Cell types are traditionally defined by morphology (Fischbach and Dittrich, 1989) but with connectomes it makes sense to define them by connectivity as well. One natural way to group cells is to represent their connections by a vector of connection strengths to various other types. These vectors can be grouped by distance to create a dendrogram, grouping together cells with similar connectivities and separating cells with very different connection patterns. An example is shown in Figure 2.

Another natural representation of a connectome is as an instance of a directed graph. Circuits are easier to visualize connections as a graph rather than a collection of tables, even if the information is the same. In the circuits reconstructed so far, nervous systems are seemingly constructed of several motifs small enough to be easily visualized, including reciprocal connections and small loops. These graphs may be annotated with connections weights (expressed in number of synapses).

A connectome expressed as a graph also facilitates queries defined by connectivity, such as “Find all cells of type A that connect to any instances of type B by a path of 2 hops or less.” A connectome can be loaded into a graph database such as Neo4j (Miller, 2013), and then a variety of graph query languages (Wood, 2012), such as cipher or Gremlin, can be used query the data.

**Figure 3:**
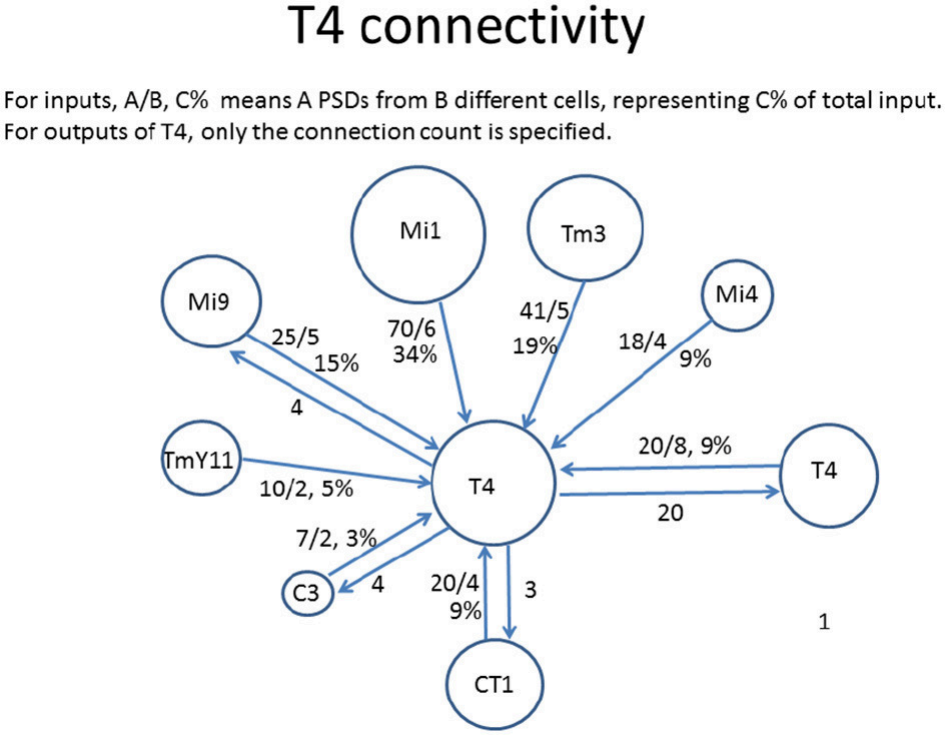
Connectivity to and from each T4 cell in *Drosophila*, as shown by a reconstruction of 7 columns of the optic lobe (Takemura et al., 2015). The incoming strengths are indicated as A/B C%, where A is the total number of synapses to all cells of that type, B is the number of cells connected, and C is the percentage of total input (output). The outgoing strengths are the number of synapses. The area of each circle is roughly proportional to the connection strength.

One often requested graphical form is the connections to just one cell type, as shown in Figure 3. In general only the stronger connections are of interest. Also, for some purposes the connections to one instance of a cell are wanted, but in other cases it might be the average connectivity to all cells of the same type.

One of the main reasons to draw a graph, rather than a table or list, is to enable human understanding of circuit operation. It is therefore important that the display diagram be designed not only to be technically correct, but to show the information flow in a way that is easy for humans to understand. Programs that do this for arbitrary electronic circuits (Jehng et al., 1991) and directed graphs (Gansner et al., 1993) have long existed. These could perhaps be mined for ideas helpful for drawing biological networks.

**Figure 4:**
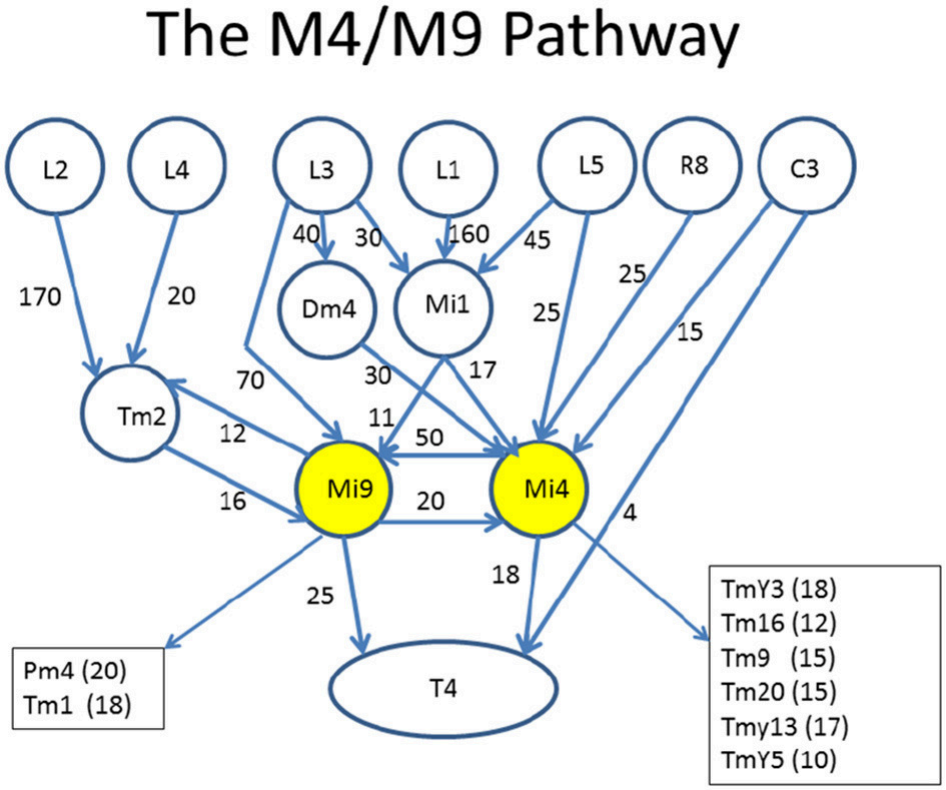
Circuits leading to Mi4 and Mi9, and hence to the motion detecting cell T4. To facilitate human understanding, the signal flow is largely uni-directional (top to bottom in this case), there are relatively few line crossings, and the edges are annotated with weights. This diagram was drawn manually, but automated and semi-automated tools to create such diagrams would be helpful. Data from Takemura et al. (2015).

An example of what is desired is shown in Figure 4. This diagram was created (manually) to highlight the role of two cell types, Mi4 and Mi9 from the medulla, in the pathways to the motion detecting cell T4. Mi4 and Mi9 have strong cross-connections, and between them receive inputs from many cells from the lamina. In particular, Mi1 is a strong contributor to both paths. The diagram is organized with inputs at the top and the T4 cell at the bottom. Only strong connections are shown, and other inputs to the T4 are ignored in this diagram.

## 4. Formal

We define “Formal” analysis as the analysis used in formal scientific documents such as papers, theses, and proceedings. Such analysis should at least be archival and reproducible, and ideally easily extendable. A scientist who seeks to reproduce the results might wish to do so at several levels:

- Take the orginal raw data, re-reconstruct and re-analyse it.
- Take the connectome as input and write their own analysis code.
- Reconstruct another organism, then run the first papers analysis.

These options require physical access to the data, an understanding by programs and humans of how the data are structured, and ability to run the analysis. We consider each of these issues in turn.

Due to the recent introduction and rapidly evolving nature of connectome analysis, no standards are yet available, and publication of data sets and analysis code is largely handled on an *ad-hoc* basis. Another problem is that the data sets are large (often many terabytes). Thus the data are too big to publish as supplemental data to a paper, and must be archived elsewhere.

### 4.1. Formal analysis

Analyses of connectomes are varied and often complex (up to tens of thousands of lines of code). For such procedures, as data scientists are well aware, the “Methods” section of a paper is just a summary of the actual analysis performed. Details such as the resolution of ties in sorting procedures, the numerical precision of intermediate results, differences in library routines, and so on, make it almost impossible to precisely reproduce results from the methods section alone. In general (one hopes) this does not affect the main points made in the paper, nor affect the conclusions when comparing substantially different organisms. However, when connectomics advances to comparing closely related species then it will be critical to use the exact same software for both, to ensure that any differences found are the result of biology and not an artifact of slightly different computation.

There are two main approaches to this problem. One is to centralize the analysis, so all researchers are using the same program. The other is to publish the code and the access methods. Then each researcher should be able to run the analysis at their own facility, and ideally get the exact same result.

The field of genomics had similar problems. The adopted solution (at least in the USA) was a funded center, the National Center for Biotechnology Information, that both stored the data and hosted the primary analysis tools. The initial version (Wheeler et al., 2000) stored mostly genetic data but it has since expanded to hold other related items (NCBI Resource Coordinators, 2018). This helps in several ways. Two different papers, using (for example) BLAST, can be compared directly since they use the same analysis tools. Next, since the data sets and analysis tools are hosted on the same site, the network bandwidth requirements are much reduced.

Could such a centralized analysis work for connectomics? Probably not yet, since tools have not yet converged on a commonly used set. To show this, we look at a (small) subset of analyses that have been attempted, and what tools were used, based on published analysis of large connectomes, both our own and others. This is shown in Table 1. Even this subset shows that analysis tools span a wide range of methods and techniques, and most analyses so far have typically been computed in an external tool such as Matlab, R, or Python.

**Table 1:** Analysis tools as used in a selection of connectome analyses.

**Paper**	**Analysis**	**Tools used**
Wiring optimization can relate neuronal structure and function (Chen et al., 2006)	Wire length optimization	MatLab
Exploring the retinal connectome (Anderson et al., 2011)	Various	Python, Excel, Tulip (Auber, 2004), Graphvis (Ellson et al., 2001)
Wiring specificity in the direction-selectivity circuit of the retina (Briggman et al., 2011)	2 photon correlation, specificity of synapses	MatLab, ITK-SNAP, custom software
Network anatomy and *in vivo* physiology of visual cortical neurons (Bock et al., 2011)	2 photon imaging of same sample, statistics of connections	MatLab, Linux tools, custom software
A visual motion detection circuit suggested by *Drosophila* connectomics (Takemura et al., 2013)	Receptive fields	C++, Matlab, Gephi (Bastian et al., 2009)
Connectomic reconstruction of the inner plexiform layer in the mouse retina (Helmstaedter et al., 2013)	Various	Matlab, Mathematica, Amira
Synaptic circuits and their variations within different columns in the visual system of *Drosophila* (Takemura et al., 2015)	Stereotypy	C++, Matlab, Linux tools
Saturated reconstruction of a volume of neocortex (Kasthuri et al., 2015)	Additional structures (mitochondria, spines, and so on)	MatLab, AutoDesk, custom tools
A connectome of a learning and memory center in the adult *Drosophila* brain (Takemura et al., 2017a)	Poisson statistics of connections	C++, Boost, Linux tools
The complete connectome of a learning and memory centre in an insect brain (Eichler et al., 2017)	Single vs. Multi-claw	Matlab, R, and Python

*The are only examples from a much larger field of studies, and intended only to show the wide variety of tools and languages employed*.

One common analysis matches receptive fields to the circuits that compute them, such as in Briggman et al. (2011), Bock et al. (2011), Takemura et al. (2013), and Takemura et al. (2017b). These analyses can't be done with connectomes alone—they need the physical location of the input, such as the location of photoreceptors in the retina or the hexagonal grid of the flys eye. They then require a weighted sum through the network, and perhaps network signs and delays as well. Receptive fields for other modalities such as olfaction, gustation, or auditory, will be very different and require specialized analyses of their own.

Another sample analysis is that of stereotypy. To examine the limits of neural wiring accuracy, Lu et al. (2009) compared the wiring of the same neurons on the left and right sides of a mouse. Similarly, Takemura et al. (2015) examined a particularly stereotyped system, the medulla of *Drosophila*, comparing each of 7 nearly identical columns against each other, using a detailed statistical model to try to separate the different potential causes of differences—differences in biology of pre- and post-synaptic counts, and reconstruction errors. In both studies, one of the main goals was to measure the rate of biological differences and errors, by manually re-examining all differences between the sides and/or columns. This is unlikely to be a common operation while reconstruction is limited to a single specimen, since such a crystal-like repetition of circuits is not expected in most parts of the brain. It will become more common, however, as comparisons of connectomes across multiple animals are tried, once increased throughput makes this practical.

Another very specific analysis is that of randomness of a specific set of connections. This was examined in the visual cortex of the mouse (Bock et al., 2011), and the olfactory system of *Drosophila* larva (Eichler et al., 2017) and adult (Takemura et al., 2017a), respectively. In each case, preliminary analysis showed no obvious pattern of connectivity between certain classes of input cells and the output neurons. However, to back up this apparent randomness, a detailed statistical model was required, and then the circuit compared to this model, generating p values, statistical powers, and so on. While the basic problem of modeling seemingly random connections is likely to re-occur, the details of each computation make it unlikely that the exact computations can be re-used.

These examples of the various and sundry analyses used show that it is unlikely that any reconstruction tool could perform all, or even most, of the analyses needed after reconstruction. Therefore, we find no practical alternative to the use of external tools, so the challenge is to make the use of such tools convenient, transparent, and reproducible. Transparency is the easiest to address, with the analysis code posted on a publically available site such as GitHub (Blischak et al., 2016) or included as supplementary data.

### 4.2. Formal data storage

More difficult, perhaps, is storing the connectome data itself in a reproducible and archivable way.

Formal analysis is based on, and analyzes, many different products of the reconstruction process. In all reconstruction techniques to date, EM, optical, or genetic, the raw data is large, and requires significant processing to generate a connectome. While here the discussion concentrates on EM, the same principles will apply if other modes of analysis are used.

In order of decreasing size, the data used in EM connectivity analysis is:

- The source EM images.
- The aligned, stitched, and normalized image stack.
- The segmentation of the volume into neurons.
- Skeletons, which are a list of 3-D points and line segments that approximate the full and typically complex shape of the neuron. These are typically formatted as SWC^1^ files (Carnevale and Hines, 2006) with an additional list of synapse locations.
- A graph, with neurons as nodes and synapse counts as weights.

Reproducing or extending an analysis will require using one or more of these representations. The raw source EM images are probably not of general interest, and “Contact the authors” probably suffices. The aligned, stitched, and normalized images form the source for machine segmentation and human proofreading. These could be made available as a stack of images, with the main problem not the technical storage but instead who will maintain (and pay for) such storage over archival lifetimes. As of mid-2018, the cheapest cloud storage costs about $4 (US) per terabyte per month. Thus a 100 TB data set costs about $400/month to store. For an active project this is reasonable, but for a 50 year archive, the cost would be $250,000 US, or the cost of several researcher-years. Most universities and research institutions would not feel such archiving is their responsibility. Even if they did, research institutions, and their focus areas, come and go over decade-long time scales. Universities and scientific journals have longer histories, but not the budgets to pay for archival storage.

Technically, reading a stack of stored images, no matter how large, should not be problem. Smaller examples can be read by publically available software such as ImageJ (Schneider et al., 2012), or its distribution FIJI (Schindelin et al., 2012), already commonly used in neuroscience. Larger examples can be read by existing software such as BigDataViewer (Pietzsch et al., 2015), a public extension of Fiji. There are higher performance and cloud compatible solutions, such as the internal format “n5” (Saalfeld, 2017) of BigDataViewer, but the longevity of these formats has not been established, whereas a stack of images should be readable for the forseeable future.

Segmentation can be stored similarly, with more bits per pixel but much better compression, due to long runs of the same value.

Skeleton data is smaller and is commonly stored as text files. There is an existing public and funded database for this, “Neuromorpho.org” (Ascoli et al., 2007). However this does not include the synapse locations or any volumetric description, and so can only store part of the results of connectomics.

### 4.3. Making sense of the data

Acquiring the physical bits that describe a connectome is only part of the problem—the next problem is making sense of it. There are two main technical methods by which external tools can get connectome data for analysis. In the oldest method, the reconstruction software writes out the relevant data as files, normally in text formats such as JavaScript Object Notation (JSON) (ECMA International, 2017) or Extensible Markup language (XML) (Bray et al., 1997) for the connectome, and SWC for the skeletons. Then an external program can read and parse these files, then do the requested analysis. In a more modern approach, a program wishing to do analysis requests the data it needs from a reconstruction server. This has been done for CATMAID (Saalfeld et al., 2009), VAST (Berger, 2015), and DVID(Katz, this issue), three recent reconstruction tools. This method has several advantages - no export step is necessary, only the needed data is transferred, and the external analysis gets the most recent version of the data (or the requested version, if the reconstruction tool supports versioning).

Multi-decade archiving requirements are most easily met by using simple text files, combined with programs in standard languages such as C++ or Python. Such files and programs will likely be readable and usable decades from now—for example, many FORTRAN programs from 50 years ago, such as LINPACK (Dongarra et al., 1979) are still used today. Furthermore, files from multiple sources can be easily combined, and files have a much lower barrier to entry. Students anywhere in the world, by themselves, could easily download the relevant files and start analysis. A one-time file format conversion is typically a one day job for an undergraduate, whereas modifying a server to support a different set of queries can take months of an expert's time. Furthermore, if running the analysis requires connection to a server, the process has considerably more human overhead, either requiring someone to run and maintain a local instance of a server, or considerable cooperation from already busy researchers.

The approach of querying a server for connectome data, while undoubtedly convenient, has downsides for reproducibility, with exactly the same risks as references to web sites. In a decade or two, the servers may be unavailable, the queries that are supported may have changed, or the owners may have moved on and no longer remember (or care) how to run the needed servers. A number of technical fixes to this problem have been proposed, such as scientific workflows (SWFs) (Altintas et al., 2004), virtualization (Dudley and Butte, 2010), and automated build systems such as Docker (Boettiger, 2015). However, each of these has their own disadvantages and overheads, particularly when combining two or more analyses that were archived using different methods. Furthermore, the author is skeptical that these methods will remain effective over the multi-decade timescales desired for scientific reproducibility.

However, despite these drawbacks, the use of servers with queries instead of text files is technically inevitable. Text forms are not efficient enough for the bigger data sets, and with a large data set a way to get desired subsets will be needed in any event. Larger and more powerful computers will not solve this problem, as their capacity will surely be used to attack correspondingly larger problems. Therefore it is incumbent on the researchers in the field of connectomics, in the interest of scientific reproducibility, to make sure their interfaces are efficient, stable, and well-documented.

### 4.4. Formal analysis conclusions

Public and archival storage of connectomic data and algorithms remains an area for development. For now, the field is dependent on the good will of practitioners to preserve and provide access to the data they collect, and the algorithms that operate on that data. We urge that they continue to use best practices, and perhaps a concensus solution will emerge. A funded center, with storage and the most common analysis tools, seems like the long term answer. The National Center for Biotechnology Information already stores and analyzes many forms of biological data, in addition to its original charter of genetic information. It would make sense for this center, or its equivalent in other countries, to pick up the task of storing and providing access to connectomic data.

## 5. Input

The final use case is “Input,” where the connectome is used as input to another process. In general the goal of connectome reconstruction is not the connectome itself, but a mechanistic understanding of the operation of the nervous system. This involves integrating other data, obtained from other sources by other methods. This is because the EM images typically used for circuit reconstruction show the detailed shapes of cells, and the existance, location, and partners of synapses, but many details critical to the circuit and synapse operation are not visible in these images. Gap junctions and synapse models including transmitter and receptor types are the most obvious examples, but locations of ion channels, receptors and sources for neuromodulators and hormones, biochemical cascades affecting synapses, and sensor/actuator links to the sensory and motor system are needed as well.

This additional data must be generated by methods other than electron microscopy. The neurotransmitter(s) of each cell can often be determined by techniques such as RNA sequencing (Croset et al., 2018), or Fluorescent *in-situ* Hybridization (FISH) (Spencer et al., 2000). Receptors expressed by a cell can also be found by RNA sequencing, but this does not tell where each receptor is expressed. This is a particular problem in insects, where many of the main transmitters, such as acetylcholine and glutamate, have multiple different receptors, sometimes of differing sign (Osborne, 1996), and all expressed in the same cell. In the case of a single receptor and a single cell type, this problem has been approached via FISH, but techniques with higher throughput are clearly needed. A combination of multi-color labeling (Bayani and Squire, 2004), genetically identified cell lines, and expansion microscopy seems the most likely approach to resolving this. An entirely different approach (Jonas and Turaga, 2016; Tschopp and Turaga, 2018) is to reverse fit the known operation to try to find the sign, strengths, and time constants of the synapses.

Integrating this additional data with connectomes is both an opportunity and a requirement in the quest to understand the operation of the nervous system.

### 5.1. Input for simulation

One typical use for connectomes includes neural simulators such as Neuron (Carnevale and Hines, 2006), Genesis (Bower and Beeman, 2012), or Nest (Gewaltig and Diesmann, 2007), or a theoretical model of circuit operation. This seems straightforward in principal, but there are several concerns. First, there can be problems with the accuracy of extracted values. Second, the data (particularly from EM) can be too detailed, and overwhelm downstream tools. Conversely, some of the required data will still be missing, and must be supplied from other sources.

One problem is the accuracy of extracted parameters, such as the cytoplasmic resistance and the membrane capacitance. Some techniques for obtaining connectomes, such as bar-code sequencing, do not generate this information, even approximately, so it must be supplied from other sources. Even techniques that do reveal morphology of cells, such as optical or EM, are subject to errors introduced in the staining and fixing process. None of the reconstruction techniques reveal the resistivity of the cytoplasm. Membrane capacitance is well-defined, per unit area, but influences such as myelinization can change the effective value.

**Figure 5:**
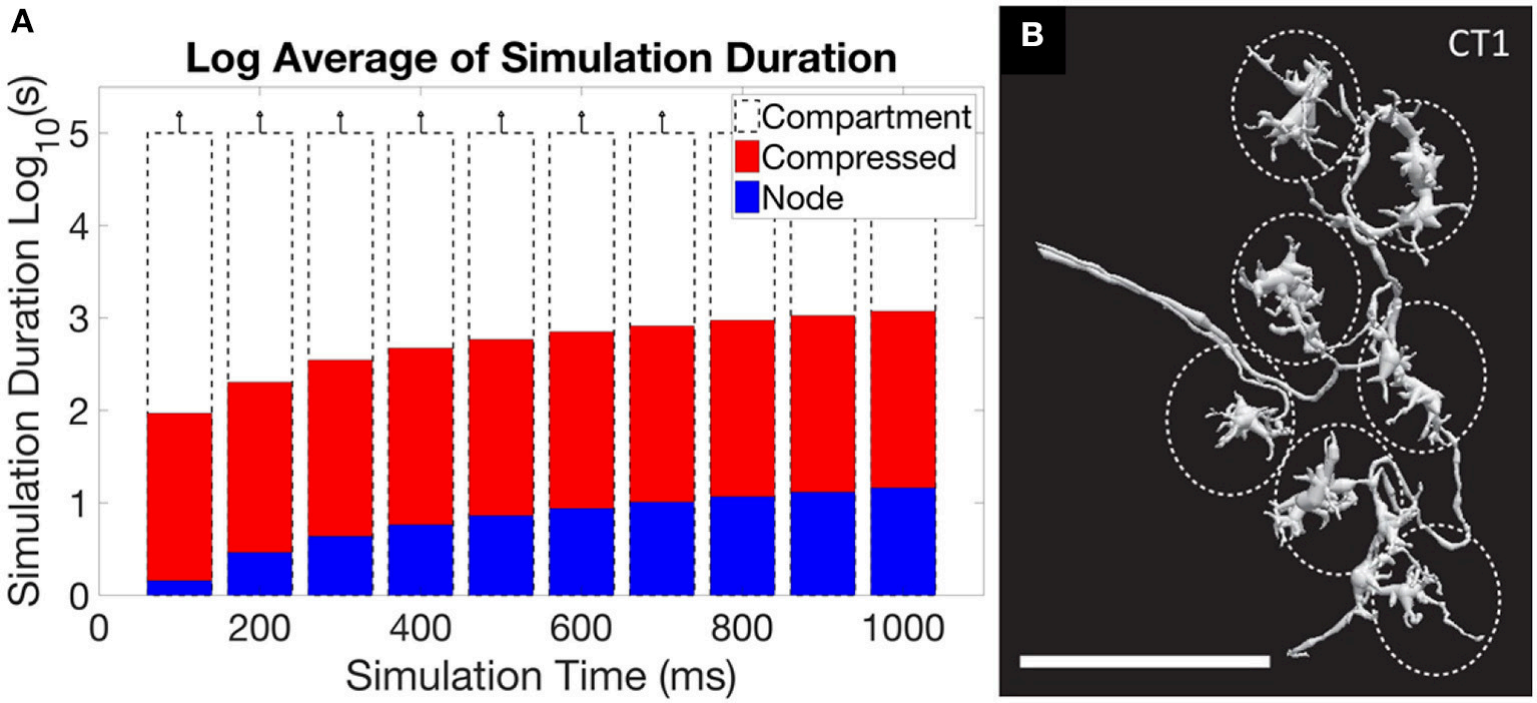
An illustration of the central problem of circuit simulation from EM reconstruction. **(A)** shows the run time for a simulation of the ON-pathway motion detection circuit from *Drosophila*, when exposed to patterns moving in the four cardinal directions, using the simulation program *Neuron*. When the full “compartment” model (one compartment per extracted segment) from the EM reconstruction was used, run times exceeded 10^5^ s, or more than one day. The “compressed” form, which keeps only the branch points of the neuron and merges all other segments, ran in minutes. The “node” model, where each neuron is represented as a single compartment, ran in seconds. For this circuit, the difference in accuracy between the representations is small (Gornet and Scheffer, 2017). **(B)** however, shows the neuron CT1, where reduction to a single node leads to incorrect results. The large size (scale bar is 10 μm) and small connecting neurites create a many-millisecond delay between the clusters defined by the dotted ellipses. If the neuron model is compressed to a single node, as is optimal for **(A)**, this delay will not be simulated correctly.

Another problem arises if simulation of extracted connectome, or a theoretical model of operation, is the goal. In these cases the models from EM reconstruction are typically much more detailed than needed, requiring intelligent reduction to get a useable representation (Gornet and Scheffer, 2017). A typical neuron reconstructed by EM has hundreds if not thousands of segments, typically represented as an SWC file. This is much more detail than required, at least when considering electrical effects, and results in impractical runtimes. Reducing the level of detail leads to orders of magnitude better execution times, and for many neurons the resulting error is acceptable. There are some neurons, however, where full reduction leads to inaccurate simulations. This problem is illustrated in Figure 5, where the first panel shows the impracticality of including all detail, but the second panel shows a case where the detail cannot be entirely ignored.

This analysis can be quantified using a simple approximation of simulation accuracy, which shows that EM produces much more detail than is likely required, but that larger neurons cannot be reduced to a single compartment. Neurons operate on roughly millisecond time scales. Compartments with much smaller time constants make solving the equations of simulation difficult (due to both the large number of compartments and the wide span of time constants) while adding little accuracy. Compartments with time constants much larger than a millisecond are easy to simulate but may be silently inaccurate. So what is in general desired is a model with time constants somewhat less than a millisecond, but not too much less. The exact tradeoff of course depends on the accuracy needed and the circuit under analysis.

Using a resistor-capacitor(RC) model to estimate time constants, the Elmore delay (Elmore, 1948) *d* of a cylinder of diameter *D*, length *L*, cytoplasmic resistivity ρ, and membrane capacitance *C*_*m*_, is

(1) $$\begin{array}{r} d=\frac{R\cdot C}{2}=\frac{1}{2}\cdot \rho \frac{4L}{\pi D^{2}}\cdot C_{m}\pi dL=\rho C_{m}\frac{2L^{2}}{D} \end{array}$$

**Figure 6:**
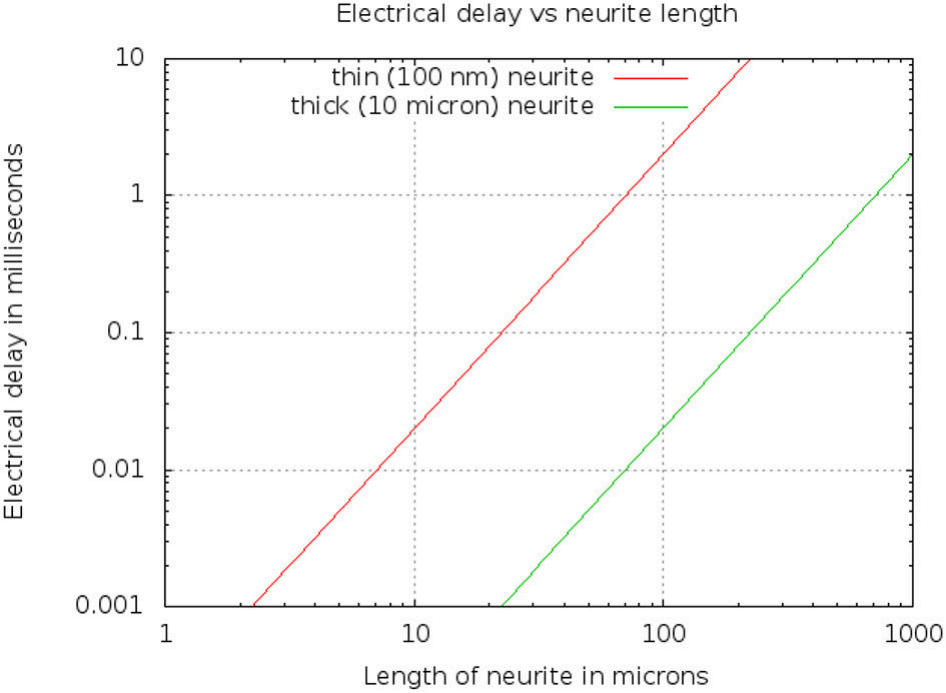
Electrical delay through a simple model of a neurite as a function of length and diameter.

Typical values are ρ = 1 ohm·m, and $C_{m}=10^{-2}$ F/m^2^. A thin branch might have a diameter *D* of 100 nm or 10^−7^m, while a very thick neurite might have a a diameter of 10μm. The resulting delays are shown in Figure 6. For example, a length *L* of 50 μm yields a delay of 0.5 millisec for a thin neurite.

Since the delay scales as *L*^2^, but only inversely as diameter *D*, this means that even a very thin branch will allow compartments 10μ in length, within which the differential delay will be less than 100μs. Conversely, long neurons (such as those 1 mm or longer) will need to be divided into compartments, even if they are very thick, to keep the differential delay under a millisecond.

This drives the requirement that the EM skeletons must be reduced (otherwise they will overwhelm simulation resources and create time-constant problems), but cannot be reduced indiscriminately (or they will not be accurate). Very similar problem have been addressed in the field of electronics when simulating circuits derived from IC layouts, and previously developed solutions (for example, Sheehan, 1999; Ionutiu et al., 2011; Reis and Stykel, 2011) can provide good starting points here.

Consider what would be needed to do a simulation of a functional unit comprising a subset of a much larger connectome. Even in a small animal such as an insect, just defining the subset of neurons to simulate is already a big task if performed manually—a typical functional circuit (say the mushroom body, the seat of olfactory learning in insects) already contains a few thousand neurons. In a vertebrate or mammal, the circuits are likely even larger. Here are some of the steps required:

- First, the user needs to pick out the relevant neurons that define a sub-circuit to simulate. This can require defining sizeable subsets from circuits containing hundreds of thousands of neurons (for connectomes currently being reconstructed as of 2018). This is already too big to do manually.
- Next, the user must decide what to do with the neurons that stick out of the volume. Outside the volume, they may not need synapses, but will need ion channels, cytoplasmic volume, and so on, to get characteristics such as time constants right.
- The user must decide which synapses to include. Since these are often detected automatically, there may be a recall-precision tradeoff in this decision. The user may wish to get as many synapses as possible, at the cost of false positives, or use only those that are certain.
- The user must decide how the synapses work. The first step is defining the neurotransmitters(s). These may be available from NeuroSeq or explicit staining, but these are different databases, maintained by different folks for different purposes, using different nomenclature.
- Next, the receptors need to be decided. Often there are multiple families of receptors (for example muscarinic and nicotinic) and then many variations on these.
- The user must decide how to drive the inputs and what outputs to measure. The neural coding used by animal brains can make this cumbersome. For example, one representation of odor in a fruit fly is believed to involve a 6% randomly sparse representation of roughly 3000 neurons. Even defining one of these patterns requires effort.
- The software must then compress the neurons down to a sensible size, small enough to simulate efficiently but not so small as to introduce significant inaccuracy.
- Finally, then the user can perform simulations to try to figure out biology, likely involving comparisons to electrophysiology and/or behavior.

To make this easier, the software that writes the simulator input should do a number of these (non-trivial) operations automatically, then format the file for the simulator concerned (perhaps Neuron, Genesis, or Nest).

An interesting problem that has not yet been seriously addressed is matching simulation results with *in-vivo* recordings made before the reconstruction. Several data sets have acquired *in-vivo* 2-photon calcium imaging of nervous system activity before *ex-vivo* reconstruction, usually with the goal of identifying some subset of cells in later images (Bock et al., 2011; Briggman et al., 2011; Lee et al., 2016). Matching simulated with measured results holds the potential of demonstrating that all relevant factors have been considered. We are quite far from this ideal currently, due to both lack of detailed knowledge of much of the cellular machinery, and limitations of current reconstructions. In particular, all existing reconstructions include many neurons that extend outside the reconstructed volume. Accurate simulation of these neurons is impossible, nor can the activity of all such neurons be adequately measured by existing techniques. Better recording techniques, increased knowledge of cellular detail, and larger reconstructions will all bring this goal closer, but it remains many years away.

Finally, large, and particularly full-animal, connectomes will drive the requirement to co-simulate with mechanical and other simulators. These will be animal and environment specific, such as acoustic simulation for animals that echolocate, hydrodynamic simulation for animals that swim, and aerodynamic simulation for animals that fly. This co-simulation will require cross-domain conversion, for example converting neural activity to muscular forces, to drive mechanical models, and converting joint angles, forces, and other sensory inputs back into neural codes. Steps in these directions have been taken by programs like AnimatLab (Cofer et al., 2010) and FlySim (Huang et al., 2014), but much more remains to be done.

## 6. Conclusions

Until recently, connectomes have been difficult and time consuming to acquire. Analysis took a comparatively small effort and was performed by the same team doing the reconstruction. Reconstruction technology, however, is rapidly improving and we are about to enter a new era. In this era, analysis rather than data collection will dominate, and the researchers doing analysis will often be distinct from those doing reconstruction. This change happened quickly in the field of genomics, and we need to plan for a similar transition in connectomics.

Along these lines, we note that the many unique analyses required to date are likely a result of our lack of understanding of the principles behind neural circuit organization. It seems likely that as more and more connectomes are analyzed, patterns of circuit organization will emerge. In the future, it is therefore possible that a standard set of analyses may suffice for most users, as is currently the case for genomics.

## Author contributions

The author confirms being the sole contributor of this work and has approved it for publication.

### Conflict of interest statement

The author declares that the research was conducted in the absence of any commercial or financial relationships that could be construed as a potential conflict of interest.

## Footnotes

^1^SWC is not an abbreviation, but the initials of the developers (Stockley et al., 1993).
